# Deficits in psychological resilience and problem-solving ability in adolescents with suicidal ideation

**DOI:** 10.1186/s13034-023-00577-z

**Published:** 2023-03-02

**Authors:** Lin Xu, Hangbin Zhang, Chendi Zhou, Zhongwen Zhang, Gen Li, Weicong Lu, Xinhe Tian, Hebin Huang, Danping Li, Robert J. Schinke, Tifei Yuan, Jie Yin, Kangguang Lin

**Affiliations:** 1 School of Psychology, Beijing Sport University, No.48 Xinxi Road, Beijing, 100084 China; 2 Shanghai Key Laboratory of Psychotic Disorders, Shanghai Mental Health Center, Shanghai Jiao Tong University School of Medicine, Xuhui, Shanghai China; 3 Department of Affective Disorder, Guangzhou Brain Hospital, The Affiliated Brain Hospital of Guangzhou Medical University, No.36 Mingxin Road, Guangzhou, Guangdong Province China; 4 School of Kinesiology and Health Sciences, Laurentian University, 935 Ramsey Lake Road, Sudbury, ON P3E-2C6 Canada; 5 School of Health and Life Sciences, University of Health and Rehabilitation Sciences, Qingdao, Shandong Province China

**Keywords:** Psychological resilience, Problem-solving ability, Grade one junior middle school students, Suicidal ideation

## Abstract

**Objectives:**

This study aimed to explore differences between psychological resilience and problem-solving ability in grade one junior middle school adolescents with and without suicidal ideation, focusing on the relationship between these factors and suicidal ideation.

**Methods:**

Ninety-nine adolescents (aged 10 to 14) were divided into Suicidal Ideation (SI, n = 49) and Non-Suicidal Ideation (NSI, n = 50) grouped by the Self-rating Idea of Suicide Scale (SIOSS). The Psychological Resilience Scale (PRS) and Tower of Hanoi task (TOH) were applied to assess psychological resilience and problem-solving ability, respectively.

**Results:**

The SI group scored significantly lower than the NSI group on PRS (*p* < 0.001) and performed more poorly on TOH than the NSI group, with more mistakes in the number of errors index (*p* < 0.001) and requiring a longer time in the task completion time index (*p* < 0.05). Among all the participants in this study, a significant negative correlation was observed between PRS and SIOSS (*r* = − 0.413, *p* < 0.01). The sub-dimensions of PRS including emotional control, family support, and interpersonal assistance were significantly negatively correlated with the SIOSS total score (*r* = − 0.361, − 0.360, − 0.382; *p* < 0.01).

**Conclusions:**

This study profiled the characteristics and differences in psychological resilience and problem-solving ability between adolescents with and without suicidal ideation. The data suggested adolescents with SI might have deficits in psychological resilience and problem-solving ability, which may serve as potential targets for suicide intervention.

## Background

Suicide causes significant trauma to families and it has also become an important social public health and mental health issue [1, 2]. Suicidal ideation is considered as an early psychological activity of suicidal behavior [3], and a prerequisite to suicide action [4]. Generally, suicidal ideation can include vague thoughts of death as well as specific thoughts about means and planning [5, 6]. Although engaging in suicidal ideation does not necessarily mean an individual will act on their contemplations, it is a strong predictor of suicide attempts. According to the 2021 China Health Statistics Yearbook, suicide has become the third leading cause of accidental injury death in the age group of 10 to 20 years old in China [7]. Adolescents are in a critical period characterized by rapid physical and psychological changes and developments [8]. Although adolescents’ independent consciousness is significantly enhanced, their psychological tolerance in the face of difficulties and setbacks remains vulnerable [9], and they are more prone to be filled with contradictions and agitation than adults [10]. Due to the weak psychological ability after suffering from life pressures or malignant events, frequent suicide attempts of adolescents has become a key issue of urgent concern [11]. Therefore, it is essential to understand factors associated with these problems in order to identify adolescents’ suicidal ideation proactively.

Researchers have found that depression, despair, and other negative emotions [12, 13], as well as substance abuse [14], stressors in life, and irrational beliefs [15], are risk factors for suicidal ideation. Meanwhile, suicidal ideation is associated with adolescents’ personality characteristics [12], parental rearing patterns [16], and family environments [1]. The integrated motivational-volitional (IMV) model of suicidal behavior has revealed that the pre-motivational phase is a prerequisite to suicidal ideation, and that individual vulnerabilities elevate risk for developing suicidal ideation when activated by the presence of the aforementioned stressors [17]. Psychological resilience could be considered as an adaptive response that can be developed as part of the process and the outcome of successfully responding to difficult or challenging life experiences, especially through mental, emotional, and behavioral flexibility and adjustment to external and internal demands [18]. As the ability to maintain mental health or recover in the face of adversity increases, psychological resilience can help individuals to overcome setbacks and promote them raise to a relatively well-adjusted state [19]. Relevant studies have also shown that psychological resilience can protect individuals from the impact of perceived pressure whilst promoting individuals to have a good ability to adapt to their environment [20, 21]. Thus, deficits in resiliency features may be associated with increased developing risk of suicidal ideation and allow for early detection and prevention of suicide in adolescents.

Meanwhile, researchers have found that high school students who exhibited suicidal ideation also show lower quality problem-solving strategies [22]. In some ways, as an important component of high-level executive functions, the comprehensive assessment of problem-solving ability includes the measurement of individual planning, inhibition and working memory, reflecting whether a person has difficulties in planning, concept formation, decision making, and cognitive flexibility [23, 24]. Therefore, using an experimental task that measures problem-solving ability can identify any difference in advanced cognition function between students with and without suicidal ideation [25].

Additionally, previous studies in this subject area have mostly focused on high school students, and studies regarding psychological resilience and cognitive characteristics of adolescents with suicidal ideation in a younger age group remain scarce. Students in grade one junior middle school (aged 10 to 14) are in the early adolescent phase with rapid growth changes, both physically and psychologically. The pressures adolescents face academically and interpersonally can easily put them in a state of agitation in which they tend to be maladjusted [8], potentially causing suicide or other extreme harming behaviors [26]. Prior studies suggested differences in their attention and cognitive style, but did not investigate their problem-solving ability. Executive dysfunction may also be associated with suicidal behavior [22]. Evidence from high school students with suicidal ideation revealed shared cognitive characteristics, such as proportionately high negative self-schema [27], attentional bias [28], and semantic processing bias towards negative emotional information [6]. Those engaging in suicidal ideation also tended to assign more negative attributions [29] and preferred taking more risks in high risk decisions, such as they make high-reward but high-risk choices [30]. New results may be obtained by comparing the problem-solving ability between adolescents with and without suicidal ideation. Therefore, this study will focus on younger adolescents with and without suicidal ideation to explore their differences in terms of psychological resilience and problem-solving ability. We hypothesized that the total score of Psychological Resilience Scale (PRS) [31] and the total score of the Self-rating Idea of Suicide Scale (SIOSS) [32] of the two groups would be negatively correlated. Compared to adolescents without suicidal ideation (NSI), adolescents with Suicidal Ideation (SI) would have weaker psychological resilience as measured by PRS, and perform worse on problem-solving ability as measured by the Tower of Hanoi task (TOH) [33, 34].

## Methods

### Participants and procedure

A total of 718 adolescents ages 10 to 14 in grade one junior middle school in Guangdong Province of China were screened by the questionnaire survey of Self-rating Idea of Suicide Scale (SIOSS). According to their SIOSS score, if the total score ≥ 12, the students were considered to have suicidal ideation. Concealment molecule ≥ 4 was considered as an unreliable measurement and excluded from the data analysis. If the total score < 12, the students were considered to have no suicidal ideation. The final number of students enrolled in the Suicidal Ideation (SI) group was 49, and 50 students without suicidal ideation were selected sampled as the Non- Suicidal Ideation (NSI) group. Among the 99 students in our study, the mean age was 12.96 (SD = 0.57) and 48.4% were male. Sample size estimation was calculated from G*power and 33 students in each group minimum were required in the current study.

The ethical approval was obtained from the Ethics Committee of Beijing Sport University (ethics committee approval number: 2022220H). After obtaining written informed consent from each student’s family designated guardian, basic information and scale data were collected from the students in grade one junior middle school completed the SIOSS online. After the questionnaire data were analysed, the students included in the SI group and NSI group additionally completed the PRS and TOH tasks. After completing the test, the researchers expressed gratitude to these students.

### Measures

#### Suicidal ideation

The Self-rating Idea of Suicide Scale (SIOSS) [32] was used to evaluate suicidal ideation. The SIOSS consists of 26 items, including 4 sub-dimensions: despair, optimism, sleep, and concealment. Each item was answered with “yes” or “no”, scored as “1” or “0”. If the total score ≥ 12, the students were judged to have suicidal ideation. Concealment molecule ≥ 4 was considered as an unreliable measurement and excluded from the data analysis. The higher the scores on despair, sleep, and optimism, the stronger the sense of despair, the poorer the sleep quality, and the more pessimistic they were. In a prior study, the Cronbach’s alpha for SIOSS was 0.742, and the Bartlett’s test of sphericity was significant [41]. Cronbach’s alpha for SIOSS was 0.906 in the present study.

#### Psychological resilience

The Psychological Resilience Scale (PRS) [31] was used to evaluate adolescent’ psychological resilience. It consists of 27 items, including 5 sub-dimensions: goal focus, emotional control, positive cognition, family support, and interpersonal assistance. Goal focus refers to the ability to continue to adhere to goals and concentrate on solving problems when things are difficult to handle. Emotional control refers to the ability to adjust and control emotional fluctuations and pessimism during difficulties. Positive cognition refers to the ability to view adversity dialectically and handle it with an optimistic attitude. Family support means that family members can respect and support individuals with an acceptance attitude. Interpersonal assistance means that individuals can get help or vent their negative emotions through interpersonal relationships. PRS adopts a 5-point Likert scale (1 = “completely inconsistent”, 2 = “relatively inconsistent”, 3 = “unclear”, 4 = “relatively consistent”, 5 = “completely consistent”). All five sub-dimensions and the total score were used in analysis. The higher the score, the stronger the psychological resilience. In a prior study, the Cronbach’s alpha for PRS was 0.86, and the Bartlett’s test of sphericity was significant [42]. In the present study, the Cronbach’s alpha for PRS was 0.954.

#### Problem-Solving ability

The Tower of Hanoi (TOH) task was used to evaluate the participants’ problem-solving abilities [24, 35]. The TOH task presented on a computer screen consists of 3 equally spaced vertical pegs and 4 disks of different sizes on the pegs. Above the computer screen is the target position map, below the computer screen is the participant’s operating table. The participants were trained to move the disks from the initial distribution of positions to target positions within limited moves and complete all tasks in ascending order of move-length. In this process, participants were asked to comply with the following rules: (1) only one disk can be moved at one time; (2) a smaller size of the disk must be placed on the larger disk; and (3) the number of moves in each round is limited and the manipulation is completed according to the remaining steps prompted by the computer. In the TOH task, participants needed to firstly complete two practice phase trials which included 1-move and 2-moves subtasks, then complete the formal experiment including 2–7 moves subtasks. Among these subtasks, 2–4 moves subtasks were low-difficulty tasks, and only required low cognitive ability level, while the 5–7 moves subtasks were high-difficulty tasks, requiring participants to complete it at a higher cognitive level [36, 37]. In different subtasks, if participants had any movement that deviated from the optimal strategy, the system would prompt the participants that their current movement is incorrect, and display “Wrong order, please try again”, and then the current subtask would be restarted automatically.

### Data analyses and statistics

All statistical analyses were conducted using SPSS Statistics for Windows version 25.0. Before statistical analysis, a normality test was conducted via a Skewness and Kurtosis test [43]. According to the average value of skewness (± 2) and kurtosis (± 7), all the items were normal in this study and were assumed to be distributed normally. Descriptive statistics and between-group comparisons were made using a Chi-squared test or independent *t* test as appropriate. Pearson correlation analysis was used to analyze the relationship between psychological resilience, problem-solving ability, and suicidal ideation. To compare the TOH performance (error and time) of the two groups under different task difficulty conditions, repeated measure analysis of variances (RM-ANOVA) was used with task difficulty (high/low difficulty) as a within-subject factor, and the participants’ group (SI group, NSI group) as a between-subject factor. Then, to compare the differences in TOH performance (i.e., errors, completion time, and thinking time) between the two groups, three separate RM-ANOVAs were conducted with different subtask difficulties (2–7 limited steps of moves) as a within-subject factor, and participants’ group as a between-subject factor. We also conducted post hoc comparisons with Bonferroni corrections. P values in ANOVAs were corrected using the Greenhouse- Geisser method, and the significance level of alpha was set to 0.05 (two-tailed).

## Results

### Participants’ demographic characteristics

There was no significant difference in age or parents’ educational level between the SI group and NSI group. The demographics and the comparisons between the two groups are found in Table 1.

**Table 1:** Demographic characteristics of the SI and NSI groups

	SI * (SD)*	NSI * (SD)*	*χ*^*2*^*(t)*	*p*
Gender (male%)	*n* = 21(43%)	*n* = 27(54%)	1.230	0.267
Age (years)	12.85 (0.61)	12.87 (0.44)	− 0.164	0.870
Father’s education (years)	10.21 (3.00)	10.97 (2.19)	− 1.317	0.191
Mother’s education (years)	9.82 (3.12)	9.91 (2.51)	− 0.144	0.886

### Psychological resilience comparison between SI and NSI group

Statistical test results revealed that the psychological resilience of the SI group was significantly lower than the NSI group (*p* < 0.001). The scores of emotional control, family support, and interpersonal assistance in SI group were significantly lower than those in NSI group (*p* < 0.001, *p* < 0.001, *p* < 0.001). The relevant comparison results between the two groups are shown in Table 2.

**Table 2:** Descriptive statistics and difference analysis for psychological resilience in two groups

Variables	SI (*n* = 49) *M(SD)*	NSI (*n* = 50) *M(SD)*	*t*	*p*
Goal focus	13.80 (5.93)	14.40 (7.03)	− 0.454	0.651
Emotional control	18.14 (5.33)	23.79 (5.07)	− 5.338	< 0.001
Positive cognition	11.06 (5.26)	10.31 (5.23)	0.702	0.484
Family support	16.80 (3.83)	21.21 (4.20)	− 5.407	< 0.001
Interpersonal assistance	16.24 (5.39)	22.15 (3.66)	− 6.315	< 0.001
Psychological resilience	76.04 (11.53)	91.85 (17.72)	− 5.198	< 0.001

Psychological Resilience variable is the total score of the above five subdimensions

### Correlation analysis of variables

Correlations among variables are included in Table 3. The findings revealed that suicidal ideation was negatively correlated with psychological resilience. Emotional control, family support, and interpersonal assistance were also negatively correlated with suicidal ideation. Similarly, suicidal ideation was positively correlated with the total number of mistakes, difficult sub-tasks number of mistakes made in TOH, and the completion time in difficult tasks. Interpersonal assistance was positively correlated with the thinking time before the first step of completing the difficult task. In conclusion, these results reveal that greater suicidal ideation was negatively correlated with lower psychological resilience and lower problem-solving ability.

**Table 3:** The correlation analysis between Suicidal Ideation, Psychological Resilience and TOH performance

	Easy task errors	Hard task errors	Total errors	Easy task CT	Hard task CT	Total CT	Easy task TT	Hard task TT	Total TT	Suicidal ideation
Goal focus	0.096	0.039	0.060	− 0.002	0.057	0.046	0.082	− 0.044	0.025	− 0.149
Emotional control	− 0.042	− 0.116	− 0.110	0.045	− 0.073	− 0.041	0.097	0.198	0.181	− 0.361^**^
Positive cognition	0.072	− 0.048	− 0.020	− 0.014	− 0.040	− 0.038	0.068	− 0.066	0.005	− 0.023
Family support	0.082	− 0.068	− 0.034	0.074	− 0.008	0.025	0.046	0.040	0.050	− 0.360^**^
Interpersonal assistance	− 0.024	− 0.163	− 0.145	− 0.047	− 0.053	− 0.064	0.083	0.205^*^	0.176	− 0.382^**^
Psychological resilience	0.059	− 0.112	− 0.078	0.015	− 0.035	− 0.022	0.126	0.110	0.146	− 0.413^**^
Suicidal ideation	0.075	0.384^**^	0.347^**^	− 0.007	0.204^*^	0.163	− 0.057	− 0.141	− 0.124	1

*CT* refers to completion time, *TT* refers to thinking time. ^*^p < 0.05,^**^p < 0.01

### Comparison of problem-solving ability between SI and NSI group

**Fig. 1:**
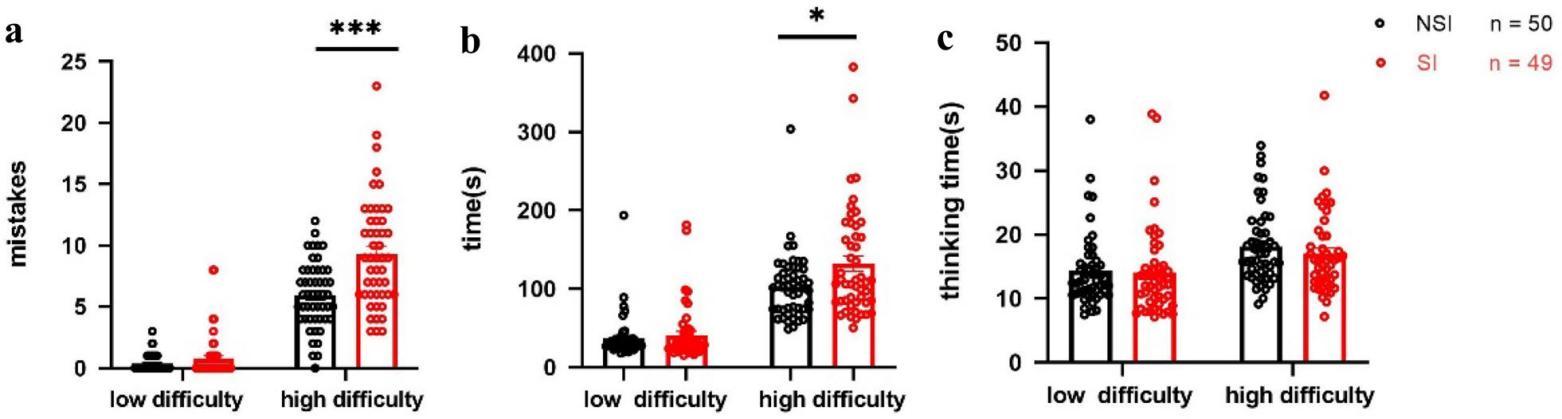
Comparison of the performance in different task difficulty between SI and NSI group. **a** Numbers of mistakes of groups in low and high task difficulty conditions. **b** Completion time of groups in low and high task difficulty conditions. **c** Thinking time before the first movement of groups in low and high task difficulty conditions. ^*^p < 0.05. ^***^p < 0.001. Error bars represent SEM

The differences in problem-solving ability between the two groups are shown in Fig. 1. On the number of errors index, the repeated measure ANOVA revealed that the main effect of task difficulty was significant (*F*_(1,97)_ = 435.151, *p* < 0.001, *η*^*2*^ = 0.818), the main effect of the group was significant (*F*_(1,97)_ = 19.148, *p* < 0.001, *η*^*2*^ = 0.165), and their interaction was significant (*F*_(1,97)_ = 19.027, *p* < 0.001, *η*^*2*^ = 0.164) (see Fig. 1a). Simple effect analysis indicated that there was no significant difference in the number of errors between two groups on the low task difficulty, but there was a significant difference in the number of errors between two groups on the high task difficulty. Specifically, the SI group showed a greater number of errors on the high task difficulty relative to the NSI group.

On the completion time index, the repeated measure ANOVA revealed that the main effect of task difficulty was significant (*F*_(1,97)_ = 176.673, *p* < 0.001, *η*^*2*^ = 0.642), the main effect of the group was significant (*F*_(1,97)_ = 489.394, *p* = 0.020, *η*^*2*^ = 0.055), and their interaction was significant (*F*_(1,97)_ = 4.497, *p* = 0.037, *η*^*2*^ = 0.044) (see Fig. 1b). Simple effect analysis indicated no significant difference in the completion time between two groups on the low difficulty task, but there was a significant difference in the completion time between two groups on the high difficulty task. Specifically, the SI group showed a greater number of errors on the high task difficulty relative to the NSI group.

On the think time index, analyses showed that the main effect of task difficulty was significant (*F*_(1,97)_ = 19.549, *p* < 0.001, *η*^*2*^ = 0.168), the main effect of the group was not significant (*F*_(1,97)_ = 0.529, *p* = 0.469), and their interaction was not significant (*F*_(1,97)_ = 0.340, *p* = 0.561) (see Fig. 1c).

**Fig. 2:**
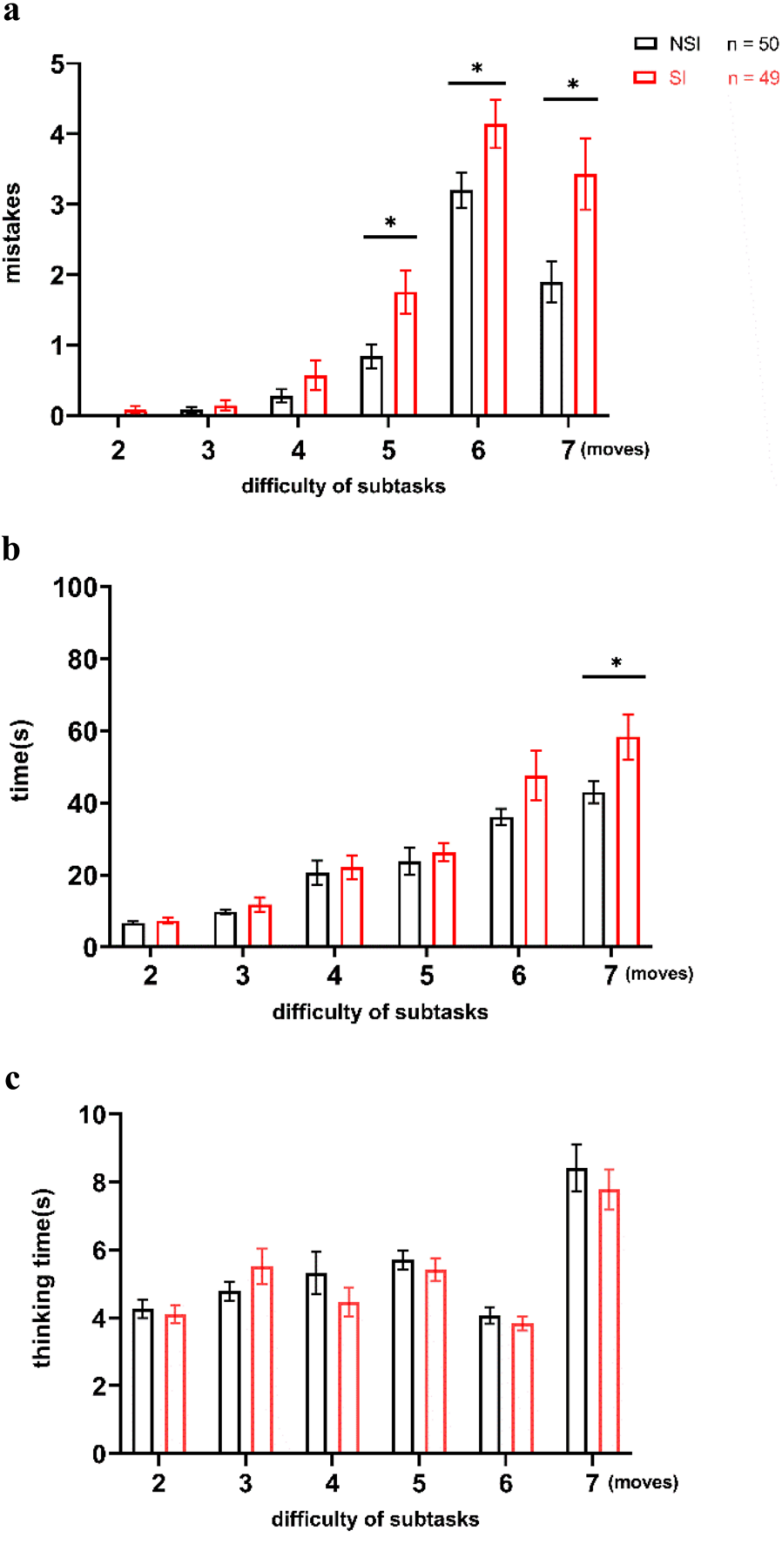
Comparison of the performance in subtasks between SI group and NSI group. **a** Numbers of mistakes of subgroups in different difficulties between two groups. **b** Completion time of subgroups in different difficulties between two groups. **c** Thinking time before the first movement of subgroups in different difficulties between two groups. ^*^p < 0.05. Error bars represent SEM

For further detailed exploration, we separately conducted three 2 (participants’ group: SI group, NSI group) × 6 (difficulty: 2/3/4/5/6/7 moves limited) repeated measure ANOVAs for the performance of the TOH task. The performance results of the two groups in subtasks of different task difficulty conditions are presented in Table 4 and Fig. 2.

**Table 4:** Performance of the SI and NSI group in subtasks

		SI (*n* = 49) *M(SD)*	NSI (*n* = 50) *M(SD)*	*F*	*p*	*η*^*2*^
Average mistake made	2-moves	0.08 (0.34)	0 (0)	1.662	0.103	0.028
	3-moves	0.14 (0.50)	0.08 (0.27)	0.778	0.439	0.006
	4-moves	0.57 (1.47)	0.28 (0.64)	1.273	0.208	0.017
	5-moves	1.76 (2.17)	0.84 (1.16)	2.601	< 0.05	0.066
	6-moves	4.14 (2.39)	3.20 (1.79)	2.217	< 0.05	0.048
	7-moves	3.43 (3.52)	1.90 (2.03)	2.637	< 0.05	0.068
Average time spent (s)	2-moves	7.32 (5.74)	6.70 (2.84)	0.681	0.497	0.005
	3-moves	11.73 (13.50)	9.80 (4.54)	0.957	0.341	0.009
	4-moves	22.20 (23.25)	20.59 (24.25)	0.339	0.736	0.001
	5-moves	25.29 (17.37)	23.78 (26.94)	0.551	0.583	0.003
	6-moves	47.59 (48.59)	36.06 (16.24)	1.577	0.120	0.025
	7-moves	58.36 (43.94)	43.02 (21.73)	2.198	< 0.05	0.048

On the number of errors index, the main effect of difficulty (*p* < 0.001,* η*^*2*^ = 0.448), group (*p* < 0.001, *η*^*2*^ = 0.165), and the interaction of difficulty × group (*p* < 0.001, *η*^*2*^ = 0.127) were significant (see Fig. 2a). On the completion time index, there was a main effect of difficulty (*p* < 0.001, *η*^*2*^ = 0.344), and group (*p* < 0.05, *η*^*2*^ = 0.055), but no interaction (*p* = 0.169) was found (see Fig. 2b). On the think time index, we also observed a significant main effect for difficulty (*p* < 0.001, *η*^*2*^ = 0.231), but no group effect (*p* = 0.469) and interaction (*p* = 0.408) were found. Post hoc analyses revealed no significant differences between the two groups in the mistakes of the simpler subtasks (2–4 moves limited tasks), while the SI group students tended to perform significantly more poorly in more difficult subtasks relative to the NSI group students: the SI group students made more mistakes in 5–7 move limited tasks and spent longer time in a 7 moves limited task.

## Discussion

Suicidal ideation is a strong risk factor for adolescents’ suicidal behavior, and it is gradually gaining attention from scholars [6, 10, 22, 28–30]. The timely detection of differences in Identifying the psychological characteristics among adolescents with suicidal ideation might allow for early pick up these at-risk individuals and prevent the occurrence of suicidal events [8]. Currently, the situation of suicidal ideation among younger adolescents warrants attention. Increasing the understanding of psychological resilience and the cognitive characteristic of problem-solving ability among the grade one junior middle school students with suicidal ideation may contribute to supplementally relevant research relating to the characteristics of suicidal ideation, their identification, and proactive treatment. Thus, we selected the grade one junior middle school students with or without suicidal ideation as the participants, and explored the characteristics and differences in their psychological resilience and problem-solving ability, and the relationships between these two variables and adolescent suicidal ideation. Below we consider the contributions derived from the study.

Firstly, the results revealed that the psychological resilience of the suicidal ideation group was significantly poorer than the non-suicidal ideation group. Meanwhile, consistent with prior research [1, 38],  the score of psychological resilience was negatively correlated with the score of suicidal ideation, suggesting that students with high psychological resilience have less psychological distress and suicidal ideation. It also suggests that educators and parents could strengthen adolescents’ psychological resilience proactively, which might be helpful for youth development in a psychosocially challenging developmental period. The sub-dimensions of psychological resilience, such as emotion control, family support and interpersonal assistance, were significantly negatively correlated with suicidal ideation. These findings suggest that in some ways, adolescents with high suicidal ideation tend to have poor emotional control ability and less social support related to family and interpersonal aspects [39]. Within the integrated motivational-volitional (IMV) model of suicidal behavior it is posited that cognitive vulnerability factors may increase an individual’s risk of suicide [17]. Hence, if the adolescents have stronger psychological resilience, they may be better able to adapt to changes and cope better. Additionally, the integration of internal resources and interpersonal relationships are also essential when developing coping strategies to face the current predicament of suicidal ideation [40]. The present study also reveals that the subdimensions of psychological resilience and interpersonal assistance have a significant positive correlation with the thinking time before the first step of completing the difficult task of problem-solving. Following logically, the above finding also reveals that as two important protective factors of grade one junior middle school students’ suicidal ideation, the decreases in psychological resilience are positively correlated with ineffective problem-solving and vice versa, both of which can be used as an important basis for identifying ideation proactively.

Secondly, the behavioral task findings revealed that there were significant differences in the performance of a TOH task between the grade one junior school students with and without suicidal ideation, mainly in the number of errors and time to complete the task. Compared with students without suicidal ideation, the suicidal ideation group made more mistakes and took longer to complete difficult tasks. This may be due to the differences in the executive function of the two groups of students. Precisely, the students with suicidal ideation seem to repeatedly insist on previous wrong operations when solving a certain path. These same students may also have difficulty in planning and cognitive flexibility, resulting in higher error rates and longer completion time. This finding is consistent with previous research [22], that the quality of problem-solving strategies of students in the suicidal ideation group is significantly lower than the non-suicidal ideation group. Our study also revealed that the total number of errors made in completing problem-solving tasks and the completion time in high difficulty subtasks were significantly positively correlated with the total score of SIOSS. Hence, the present study suggests that the cognitive differences in more difficult tasks between the two group students cannot be ignored. The present study further illustrates the importance of testing the problem-solving ability of students with suicidal ideation in early adolescence in order to subsequently intervene and support effective problem-solving skills, furthering resilience as a buffering strategy. Our study combined measures of questionnaire and behavioral task outcomes and showed that there were significant differences in psychological resilience and problem-solving ability between grade one junior middle school students with and without suicidal ideation. The decrease of psychological resilience and the defect of problem-solving ability were statistically associated with adolescents’ suicidal ideation. This may reflect the risk of suicidal ideation in younger adolescents and early exploration of cognitive differences in this area may be used as a means of identifying or predicting suicidal ideation. Further studies to investigate if improving junior middle school students’ psychological resilience and problem-solving ability may help alleviate their suicidal ideation. Hence, it is necessary to consider the cognitive characteristics of psychological resilience and problem-solving ability when assessing and identifying adolescents’ suicidal ideation, and to focus on the relationship of these factors to suicidal ideation in treatment and future research. Such investigations would also provide theoretical and empirical support for departments in ministries of education and mental health to carry out the prevention of suicidal ideation.

## Limitations

The present study has its limitations. Firstly, results of the present study may have limited generalizability, considering that this study adopted a sample survey of middle school students in China. The findings cannot be generalized to the broader adolescent population across nations, without further multinational studies. Second, in terms of causality, this research is a cross-sectional study, which can only be used to describe the characteristics and difference on psychological resilience and problem-solving ability of junior middle school students, as well as the relationship between suicidal ideation, psychological resilience, and problem-solving ability. This study cannot be used to determine causal relationships, vertically. Future researchers can carry out longitudinal research to delineate the causal factors of adolescent suicidal ideation. Thirdly, in our study, behavioral results revealed different problem-solving characteristic of adolescents with or without suicidal ideation. However, we did not take more sophisticated equipment to explore the difference in physiological mechanisms within the SI and NSI groups. Future researchers can explore this aspect more deeply.

## Conclusions

This study extends prior research by examining psychological and cognitive variables, such as psychological resilience and problem-solving ability through grade one junior middle students with or without suicidal ideation. This study suggests adolescents with SI may have deficits in psychological resilience and problem-solving ability, which may be good indicators and serve as potential targets for intervention in relation to suicidal ideation. It is our fervent hope that scholars continue to investigate broader cognitive and psychological factors to improve treatment and prevention of suicidal ideation in adolescents. In so doing, the intention would be to foster resilience and broader wellbeing in adolescents during a formative and potentially fragile developmental stage.

## Data Availability

The analytical database of this study can be obtained from the corresponding author under reasonable request.
